# *ADIPOQ* Variants rs1501299 and rs3774261 and Hypoadiponectinemia in Obese Women with PCOS: Genetic and Metabolic Interactions

**DOI:** 10.3390/life16010024

**Published:** 2025-12-23

**Authors:** Intissar Ezzidi, Sameh Sarray, Mahmoud A. Alfaqih, Nabil Mtiraoui

**Affiliations:** 1 Laboratory of Human Genome and Multifactorial Diseases (LR12ES07), Faculty of Pharmacy of Monastir, University of Monastir, Monastir 5000, Tunisia; ezzidi.intissa22@gmail.com; 2 College of Medicine and Health Sciences, Arabian Gulf University, Manama 329, Bahrain; samehmss@agu.edu.bh (S.S.); mahmoudmaf@agu.edu.bh (M.A.A.)

**Keywords:** association, PCOS, obesity, variants, ADIPOQ, hypoadiponectinemia

## Abstract

**Background:** Hypoadiponectinemia, a metabolic hallmark of obesity, is common in polycystic ovary syndrome (PCOS) yet the association of variants in the *ADIPOQ* gene with obesity in PCOS remains uncertain. To investigate whether *ADIPOQ* variants are associated with obesity in PCOS in relation to circulating adiponectin levels, and whether integrating genotypes, adiponectin, and a polygenic risk score (PRS) improves risk stratification. **Methodology:** In 324 Tunisian women with PCOS, classified as obese or non-obese by WHO criteria, serum adiponectin was measured, and nine *ADIPOQ* variants were genotyped using TaqMan assays. Associations with obesity were assessed using logistic regression, gene phenotype interaction analysis, and models incorporating a PRS; epistasis, QTL, and diplotypes were also evaluated. **Results:** Adiponectin levels were significantly lower in obese women and modestly predicted obesity (AUC = 0.605). Variants rs1501299 and rs3774261 were significantly associated with obesity under recessive models (OR up to 5.18, 95% CI [2.32–11.56], *p* = 7.14 × 10^−5^). Risk genotypes and haplotypes correlated with reduced adiponectin and increased obesity risk, with adiponectin levels significantly associated with the genotype–obesity relationships. A combined model including adiponectin, the two variants, and PRS outperformed single predictors. **Conclusions:** *ADIPOQ* rs1501299 and rs3774261 are associated with obesity in women with PCOS, with this association demonstrating a specific relationship with reduced adiponectin. Integrating genetic and biochemical markers improves metabolic risk profiling and supports personalized management.

## 1. Introduction

The interplay between genetic predisposition and metabolic dysfunction is a cornerstone of modern endocrinology, with Polycystic Ovary Syndrome (PCOS) serving as a prime example of this complex relationship. PCOS is a prevalent and complex reproductive endocrinopathy that occurs in 6–15% of women of reproductive age [1,2]. In Tunisia, its prevalence is estimated at approximately 12%, underscoring PCOS as a notable public health concern [3]. Indeed, it remains one of the leading causes of anovulation and infertility [4]. PCOS is characterized by a complex interplay of genetic and environmental factors, including insulin resistance (IR) [5,6].

Rotterdam criteria define PCOS by the presence of two of the following: hyperandrogenism, polycystic ovarian morphology, and ovulatory dysfunction [7]. Although not included within the diagnostic criteria, PCOS is frequently associated with metabolic disturbances including obesity, IR, hyperinsulinemia, and type 2 diabetes (T2D) [8,9,10].

Obesity, particularly visceral adiposity, aggravates both metabolic and reproductive complications in PCOS, resulting in increased IR, compensatory hyperinsulinemia, and impaired lipid metabolism [11,12].

Adipose tissue, formerly considered a passive energy reserve, is now recognized as a dynamic endocrine tissue that secretes a range of cytokines, known as adipokines or adipocytokines with key roles in metabolic homeostasis and inflammation [13].

Adiponectin, the most abundant adipocytokine, is predominantly secreted from white adipose tissue and circulates as multimeric complexes with varying molecular weights and biological functions [14,15,16]. It enhances insulin sensitivity, exerts anti-inflammatory effects, and modulates glucose and lipid metabolism [17,18]. Consequently, hypoadiponectinemia is a hallmark of obesity and metabolic syndrome and is linked to the pathogenesis of IR, and T2D [19,20].

In line with adiponectin’s centrality to PCOS pathophysiology, composite adipokine indices, specifically the adiponectin-to-leptin and adiponectin-to-resistin ratios, have been reported as useful predictors of PCOS [21]. These observations reinforce the clinical utility of using adiponectin levels or the broader adipokine milieu for risk stratification in PCOS.

The adiponectin-encoding gene, *ADIPOQ*, is located on the long arm of chromosome 3 in the 3q27 region, [22,23] and given its metabolic functions, the *ADIPOQ* gene has been a major candidate for investigating the genetic underpinnings of PCOS. However, the current state of the research is marked by controversy and diverging hypotheses. While some studies in Asian populations have suggested that the T allele of the *ADIPOQ* variant rs1501299 may offer protection against developing PCOS, other research has failed to find a direct association with the syndrome itself, instead linking *ADIPOQ* variants to metabolic sub-phenotypes like insulin resistance within PCOS cohorts [24,25,26,27]. These discrepancies highlight a critical gap in our understanding, suggesting that the influence of *ADIPOQ* may be context-dependent, varying with ethnicity, the specific clinical phenotype being examined (e.g., PCOS risk vs. obesity within PCOS), and potential interactions with other genetic and environmental factors.

To address this gap, our study investigated nine *ADIPOQ* variants in a Tunisian cohort of women with PCOS. We aimed to investigate the relationship between *ADIPOQ* genetic variations and obesity within this syndrome with a specific focus on the association with circulating adiponectin levels. To achieve this goal, we assessed the associations between *ADIPOQ* variants and obesity status within PCOS, evaluated their relationships with obesity-related traits, quantified variant–adiponectin associations, and examined the association between adiponectin, *ADIPOQ* genotype, and obesity phenotypes in PCOS. The results of this study will not only bridge a critical gap in understanding the genetic architecture of metabolic dysfunction in PCOS but also underscore the potential of *ADIPOQ* genotyping for personalized risk stratification, paving the way for novel therapeutic strategies targeting the adiponectin pathway in this prevalent endocrine disorder.

## 2. Methodology

### 2.1. Study Participants

This cross-sectional study numbered out in accordance with the guidelines of the Declaration of Helsinki [28] and was approved by the local ethics committee of Farhat Hached Hospital, (approval number: 35220228). A total of 324 adult women (≥18 years) diagnosed with PCOS were enrolled consecutively from the Endocrinology and Gynecology outpatient clinics of Farhat Hached University Hospital, Sousse, Tunisia between March 2025 and July 2025, and written informed consents were signed from all participants.

The diagnosis of PCOS adhered to the Rotterdam criteria, which require the presence of at least two out of the following three features: (1) oligo-ovulation or anovulation, (2) Polycystic Ovarian Morphology (PCOM) on ultrasound, and (3) hyperandrogenism (clinical and/or biochemical) [7,29]. All women diagnosed with PCOS in this study exhibited PCOM on transvaginal ultrasound. In the overall cohort, 89.5% presented with oligo amenorrhea and 92.0% with hyperandrogenism, while PCOM was present in 100% of participants by design. The distribution of Rotterdam criteria was similar between BMI subgroups (oligo/amenorrhea was observed in 88.7% of non-obese versus 91.9% of obese women with PCOS and clinical and/or biochemical hyperandrogenism in 91.6% versus 93%, respectively).

Only PCOS women who were instructed to make lifestyle modifications as their first-line management strategy were invited to participate in the study. Data related to the details of the lifestyle modification program (diet, exercise, and sleep hygiene education) were not collected. Exclusion criteria were (1) other etiologies of hyperandrogenism or ovulatory dysfunction (e.g., androgen-producing tumors, nonclassical adrenal hyperplasia, hyperprolactinemia, active thyroid disease, and Cushing’s syndrome; (2) current use of medications known to affect metabolic or hormonal parameters (e.g., metformin, oral contraceptives, semaglutide, glucocorticoids, or lipid-lowering agents) within the 3 months prior to enrollment; and (3) women with extremely low (below 20 kg/m^2^) or high (exceeding 40 kg/m^2^) body mass index (BMI), prespecified to reduce potential confounding from undiagnosed co-morbidities often associated with underweight or morbid obesity.

### 2.2. Data Collection and Anthropometry

Demographic and biochemical data were collected from all participants including personal and hypertension, hyperlipidemia, and diabetes family history. All anthropometric measurements were performed by trained personnel following standardized protocols to ensure consistency and accuracy. Weight was measured to the nearest 0.1 kg using calibrated electronic scales (daily calibration), and height was measured to the nearest 0.1 cm using a stadiometer, with participants barefoot and wearing minimal clothing. Measurements were performed in the fasting state during early morning visits. BMI was calculated as weight (kg) divided by height squared (m^2^). Participants were subdivided into two groups based on their BMI: non-obese (body mass index (BMI): 20–29.99 kg/m^2^; n = 238), and obese (BMI 30–39.99 kg/m^2^; n = 86) subjects, in accordance to the World Health Organization (WHO) criteria [30].

### 2.3. Biochemical Analysis

Fasting blood samples were obtained after an overnight fast of at least 10–12 h. For in women with PCOS who had regular menses, sampling was conducted during the early follicular phase (between 2 and 5 days of the menstrual cycle). For women with menstrual disturbances (oligo-ovulation, and anovulation) sampling was not restricted to any specific time frame.

Blood was collected into specific vacutainer tubes based on the required assay: sodium fluoride/potassium oxalate tubes for fasting plasma glucose (FPG); ethylene-diaminetetra-acetic acid (EDTA)-containing tubes for glycosylated hemoglobin A1c (HbA1c) and DNA extraction, and serum separator tubes for lipid profiles, hormones, and adiponectin measurements.

FPG and lipid profiles (total cholesterol, triglycerides, HDL-C, and LDL-C) were determined using standard enzymatic colorimetric methods on an automated chemistry analyzer (Cobas c311, Roche Diagnostics, Mannheim, Germany). Hormonal parameters (FSH, LH, total testosterone, estradiol, and progesterone) and fasting insulin were measured using electro-chemiluminescence immunoassays on an automated analyzer (Cobas e411, Roche Diagnostics, Mannheim, Germany). All automated assays showed intra- and inter-assay coefficients of variation < 5%.

Serum total adiponectin was quantified in duplicate using a solid-phase sandwich Enzyme-Linked Immunosorbent Assay (ELISA) kit (Quantikine^®^ Human Total Adiponectin/Acrp30, R&D Systems, Minneapolis, MN, USA, Cat. DRP300). The assay had a sensitivity of 0.246 ng/mL and intra- and inter-assay coefficients of variation of <5% and <7%, respectively. Homeostasis model assessment of IR (HOMA-IR) was calculated as FINS (μU/mL) × FPG (mmol/L)/22.5.

### 2.4. Variants Genotyping

Total genomic DNA was isolated from peripheral blood leukocytes using the salting-out method [31]. Nine variants, namely rs16861194, rs17300539, rs266729, rs822395, rs822396, rs2241766, rs1501299, rs2241767, and rs3774261 were selected based on their minor allele frequency (MAF) > 1% in Caucasian populations (and their reported clinical relevance in metabolic and reproductive disorders within the Middle East and North Africa (MENA) region [21,32]. Allele frequency data and linkage disequilibrium patterns were verified using the LDhap and LDmatrix tools within the National Institutes of Health (NIH) LDlink (https://ldlink.nih.gov/tab=ldmatrix?tab=home, accessed on 25 July 2025) and by querying the Data Browser of the All of Us Research Hub (https://databrowser.researchallofus.org/snvsindels, accessed on 25 July 2025) using the search reference sequence (RefSeq or rs number) of *ADIPOQ* variants for relevant ancestry groups.

Genotyping was carried out by employing the allelic discrimination method with VIC-/FAM-labelled probes (Applied Biosystems, Dubai, UAE). The following TaqMan assay IDs were used for each variant: C__33187775_10 (rs16861194), C__33187774_10 (rs17300539), C___2412786_10 (rs266729), C___2910317_20 (rs822395), C___2910316_10 (rs822396), C__26426077_10 (rs2241766), C___7497299_10 (rs1501299), C__26426076_10 (rs2241767), and C__27479710_10 (rs3774261).

The reaction was performed at 20 µL volume on a Step OnePlus system following the manufacturer’s instructions (Applied Biosystems). The reproducibility of genotyping was assessed through retesting blinded samples; the concordance consistently exceeded 99%.

### 2.5. Statistical Analysis

A post-hoc power analysis was conducted using G*Power software (version 3.1.9.7) to evaluate the study’s capacity to detect genetic associations with obesity. The analysis utilized the z-test family by logistic regression with the following input parameters: two-tailed test, alpha error probability of 0.05, and power = 80%. The minimum effect size was calculated by deriving the proportion of risk alleles in the control group (p_1_) from the MAF obtained from public databases (LDlink in pooled populations of Utah Residents with Northern and Western European Ancestry (CEU), Toscani in Italia (TSI), and Iberian population in Spain (IBS), which are the genetically closest to the Tunisian population). The proportion in the case group p_2_ was calculated based on the estimated Odds Ratio (OR) and p_1_ using the standard formula: p_2_ = OR × p1/1 − p_1_ + (OR × p_1_). An a priori estimation indicated that a total sample size of approximately 290 participants was required to detect an OR of 2.0 with 80% power (assuming MAF = 0.280), which is satisfied by our total cohort of 324 women.

Statistical analyses were performed using SPSS Statistics for Windows, Version 29 (IBM Corp., Armonk, NY, USA) and R software version 4.5.1 (R Foundation for Statistical Computing, Vienna, Austria). Differences in clinical and biochemical markers between non-obese women with PCOS and obese women with PCOS groups were assessed after testing distributional assumptions with the Shapiro–Wilk test. Clinical characteristics were reported as median and interquartile ranges (25th–75th). To compare quantitative variables, the Mann–Whitney U test was used.

Effect sizes were summarized as Cohen’s d to quantify standardized mean differences, independent of sample size. Model selection relied on the Akaike Information Criterion (AIC), with the model minimizing AIC considered to provide the best balance between goodness of fitness and parsimony.

We computed the area under the receiver operating characteristic curve (AUC) to quantify overall model discrimination, with higher values indicating better distinction between positive and negative cases.

To validate the categorical BMI classification used (non-obese BMI: 20–29.99 kg/m^2^, and obese BMI: 30–39.99 kg/m^2^) and address potential confounding from the inclusion of overweight individuals (BMI 25–29.99 kg/m^2^) in the non-obese category, we performed a preliminary analysis using BMI as a continuous variable in linear regression analysis (JMP Clinical 18). This approach examined whether the interaction between clinical variables, *ADIPOQ* variants, and obesity risk varied across the BMI range (25–29.99 kg/m^2^) within the non-obese group.

The allelic and genotypic frequencies were calculated utilizing the gene-counting method on the SNPassoc R package (version 2.1.2). The Hardy–Weinberg equilibrium (HWE) for each variant was estimated by a Chi-squared test, also performed with the SNPassoc R package (version 2.1.2).

Genetic association analysis was performed using binary logistic regression, adjusting for potential confounding factors (age, LH, FSH, progesterone, estradiol, and testosterone) under four genetic models (additive, codominant, dominant, and recessive). The non-obese PCOS women served as the reference for calculating odds ratios. Forest plots showing odds ratios results were generated using the “meta-R package” (version 8.2-1).

Linkage disequilibrium (LD) and haplotype association analyses were performed using the SHEsisPlus software (http://shesisplus.bio-x.cn/SHEsis.html, accessed on 26 July 2025). Spearman correlations between *ADIPOQ* tag-variants or tag-haplotypes with adiponectin levels in obese women with PCOS were conducted using a correlation model from the SNPassoc R package (version 2.1.2) and the haplo-score function in the haplo.stats R package (version 1.9.7).

Cramér’s V was calculated to assess the strength of association between categorical variables, reported as a standardized effect size from 0 (no association) to 1 (perfect association). To evaluate gene–gene interactions (epistasis), multifactor dimensionality reduction (MDR) was applied as described by Leem and Park (2017) [33] using the open-source MDR software package (v3.0.2). The above statistical figure is useful for the identification of combinations of variants that collectively influence disease susceptibility.

Polygenic risk score (PRS) was calculated to estimate the combined association of multiple genetic variants with obesity risk. The PRS was constructed as a weighted sum of risk alleles. Variants and their corresponding weights (effect sizes) were derived from the current study’s logistic regression analysis. The PRS was calculated using the formula: PRS = ∑(β × G), where β represents the weight (log-odds ratio) of each variant and G is the genotype (coded as 0, 1, or 2) representing the number of risk alleles carried by the individual. This process generates a single, continuous score reflecting an individual’s combined genetic association with obesity.

A quantitative trait locus (QTL) analysis was performed to identify genetic variants associated with the risk of obesity by logistic regression models.

All analyses were two-tailed, with α = 0.05.

Multiple testing corrections were selected based on data type. The Benjamini–Hochberg False Discovery Rate (FDR) was used for clinical comparisons to account for physiological correlations, avoiding the overly conservative Bonferroni method. However, the stricter Bonferroni correction was applied to genetic associations and gene–phenotype interactions to rigorously control the family-wise error rate.

## 3. Results

### 3.1. Clinical Predictors of Obesity in PCOS

The results, from linear regression analysis, revealed no significant discrimination between normal-weight (BMI 20–24.99 kg/m^2^) and overweight (BMI 25–29.99 kg/m^2^) PCOS women regarding clinical variable levels (Supplementary Table S1) or *ADIPOQ* variant associations (Supplementary Table S2).

To evaluate differences of clinical and biochemical markers between non-obese PCOS and obese women with PCOS, data distribution was assessed using the Shapiro–Wilk test. All variables demonstrated a non-normal distribution (Supplementary Figure S1). Accordingly, the Mann–Whitney U non-parametric test was applied to compare groups. No significant differences were observed in most clinical and biochemical parameters between non-obese and obese PCOS women after FDR Benjamini–Hochberg adjustment (Table 1), except for adiponectin levels, which were significantly decreased in the obese group (FDR-adjusted *p* = 0.030) (Table 1).

To determine which clinical, biochemical, and hormonal variables had the potential to discriminate between obese and non-obese PCOS women, we performed binary logistic regression (Table 2). Most variables had limited discriminative capacity, with AUC values between 0.476 and 0.605. HDL showed a nominally significant association (AUC = 0.601, nominal *p* = 0.005), but this did not remain significant after FDR-adjustment (FDR-adjusted *p* = 0.075). Adiponectin as a single predictor demonstrated modest discriminatory power for obesity status (AUC = 0.605), indicating its limited utility when used in isolation.

Adiponectin levels significantly differed between obesity groups, with adiponectin levels significantly lower in obese PCOS women compared to non-obese counterparts (OR = 1.15, 95% CI (1.05–1.27); FDR-adjusted *p* = 0.006) confirming the inverse relationship between adiponectin and obesity status. Although the effect size was moderate (Cohen’s d = 0.37), the Akaike information criterion (AIC) indicated that adiponectin was the best- fitting predictor. These findings indicate that adiponectin is the most relevant biochemical marker of obesity status in PCOS.

### 3.2. Minor Allele Frequencies Associations and Linkage Disequilibrium Mapping

This investigation evaluated the relationship between nine *ADIPOQ* variants rs16861194, rs17300539, rs266729, rs822395, rs822396, rs2241766, rs1501299, rs2241767, rs3774261, and PCOS–obesity risk in a cohort of Tunisian women, with allele and genotype distributions presented in Table 3. All variants conformed to Hardy–Weinberg equilibrium within the PCOS group (Table 3).

**Figure 1:**
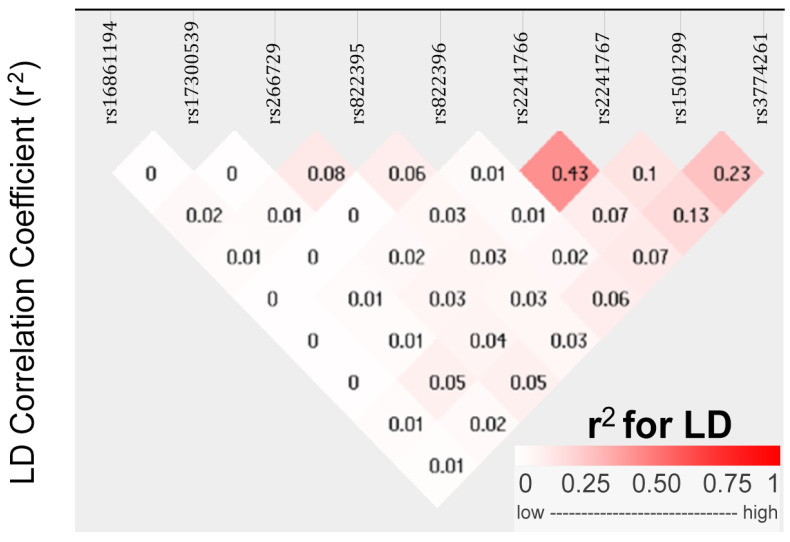
Linkage disequilibrium (LD) plot between nine *ADIPOQ* variants in Tunisian women with PCOS. Diamonds display pairwise r^2^ values between variants. The value for each variant pair is shown inside the diamond, and shading from white to dark pink indicates increasing LD according to the color scale (0–1) shown below the plot. The r^2^ is a measure of alleles correlation for two genetic variants. High r^2^ (0.8–1.0) indicates strong correlation between alleles at two variants, whereas low r^2^ (0–0.2) reflects weak or no correlation.

To understand the genetic architecture of *ADIPOQ* variants and their association with obesity in PCOS, we conducted linkage disequilibrium (LD) mapping and single-variant association studies (Figure 1). The degree of LD between different variant pairs is presented in both r^2^ values and progressive pink color scale. The r^2^ value quantifies the correlation of alleles between for two genetic variants, ranging from 0 to 1, with high LD indicated by r^2^ values between 0.8 and 1, and low LD indicated by r^2^ values between 0 and 0.2. Pairwise r^2^ values are illustrated in diamonds. LD mapping of the nine *ADIPOQ* variants revealed a haplotype block characterized by low to moderate pairwise correlations. The strongest LD was observed between rs2241766 and rs2241767 (r^2^ = 0.43), whereas correlations involving rs1501299 and rs3774261 were modest (e.g., r^2^ = 0.23).

### 3.3. Association of ADIPOQ Alleles and Genotypes with Obesity in PCOS

To determine the effect of *ADIPOQ* genotypes on the risk of obesity in PCOS patients, logistic regression analyses were conducted (Table 4). Statistically significant associations of medium effect size were observed, under additive model, for two variants, rs1501299 and rs3774261 (Cramer’s V = 0.278 and 0.236, respectively), with Power > 0.8. These results suggest that these two loci are the primary *ADIPOQ* variants associated with obesity in PCOS.

For rs1501299, the dominant model yielded OR = 2.07, 95% CI (1.22–3.50), Bonferroni-corrected *p* = 0.044), while the recessive model exhibited a best model of inheritance (AIC = 356) and a stronger effect with OR = 5.18, 95% CI (2.32–11.56), Bonferroni-corrected *p* = 7.14 × 10^−5^). For rs3774261, the dominant model yielded OR = 2.13, 95% CI (1.24–3.67), Bonferroni-corrected *p* = 0.027) and the recessive is a best model of inheritance (AIC = 359.8), OR = 3.13, 95% CI (1.69–5.78), Bonferroni-corrected *p* = 1.52 × 10^−3^). These results identify rs1501299 and rs3774261 as the key variants associated with obesity in PCOS (Table 4).

### 3.4. Epistatic Interactions Between ADIPOQ Variants

**Figure 2:**
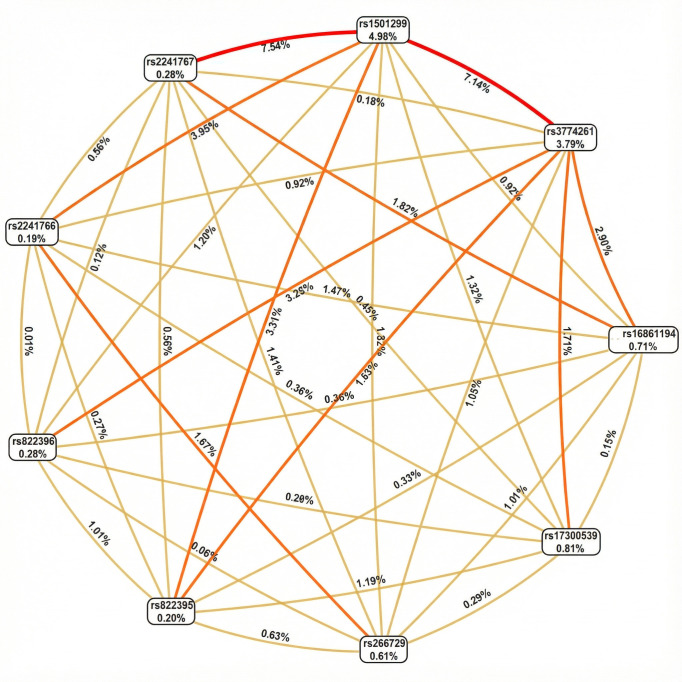
Fruchterman–Rheingold network of *ADIPOQ* variant epistasis for PCOS obesity risk. Each node represents a variant, with the percentage inside the box indicating its individual main effect strength (information gain). The lines (edges) between nodes represent pairwise epistatic interactions, with the percentage on each line quantifying the strength of that interaction. Edges color intensity reflects interaction strength (lighter/yellow = weaker interactions; darker/orange = Moderate interactions, Red = stronger interactions.

To investigate potential interactions among *ADIPOQ* variants, we applied network-based epistasis approaches (Figure 2). The analysis revealed that rs1501299 and rs3774261 had the largest individual main effects on obesity risk (4.98% and 3.79%, respectively). More importantly, these approaches identified a strong synergistic interaction between this pair (7.14%) (Figure 2). This interaction effect is substantially greater than that of any other variant pair and exceeds the individual contributions of each variant. This finding suggests that the combined effect of carrying risk alleles at both rs1501299 and rs3774261 exceeded the sum of their individual effects, highlighting a significant epistatic contribution to the genetic architecture of obesity in PCOS.

### 3.5. Correlation of Adiponectin Levels with BMI

To ascertain whether adiponectin levels alone could discriminate between obese PCOS women and non-obese women with PCOS and to evaluate their relationship with BMI, we performed receiver operating characteristics (ROC) curve and correlation analyses.

**Figure 3:**
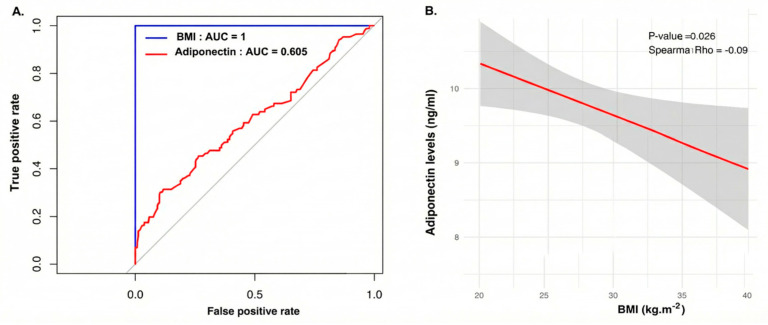
(**A**) Receiver operating characteristic (ROC) curve analysis evaluating the ability of serum adiponectin to discriminate between obese and non-obese women with PCOS. The blue line represents BMI (used as the gold standard classifier, AUC = 1.0), while the red line represents adiponectin (AUC = 0.605), indicating modest discriminatory power. The gray diagonal line indicates the reference line for random classification (no discriminative ability; AUC = 0.5. (**B**) Sperman correlation scatter plot illustrating the relationship between BMI (*x*-axis) and serum adiponectin levels (*y*-axis). The red line represents the linear regression fit, and the grey shaded area indicates the 95% confidence interval. The Spearman rho (−0.09, *p* = 0.026) confirms a significant negative correlation between adiposity and hypoadiponectinemia.

The ROC curve analysis showed that while BMI served as the reference standard (AUC = 1.0), adiponectin exhibited a modest discriminatory capacity (AUC = 0.605). This suggests that, although informative, adiponectin cannot replace BMI as a standalone classifier for obesity within this cohort (Figure 3A).

Further analysis using Spearman correlation clarified this relationship, revealing a significant negative correlation between BMI and adiponectin levels (Spearman rho = −0.09; *p* = 0.026). The regression plot confirms that as BMI increases, adiponectin levels progressively decline (Figure 3B).

### 3.6. Gene–Phenotype Interaction Analyses

**Figure 4:**
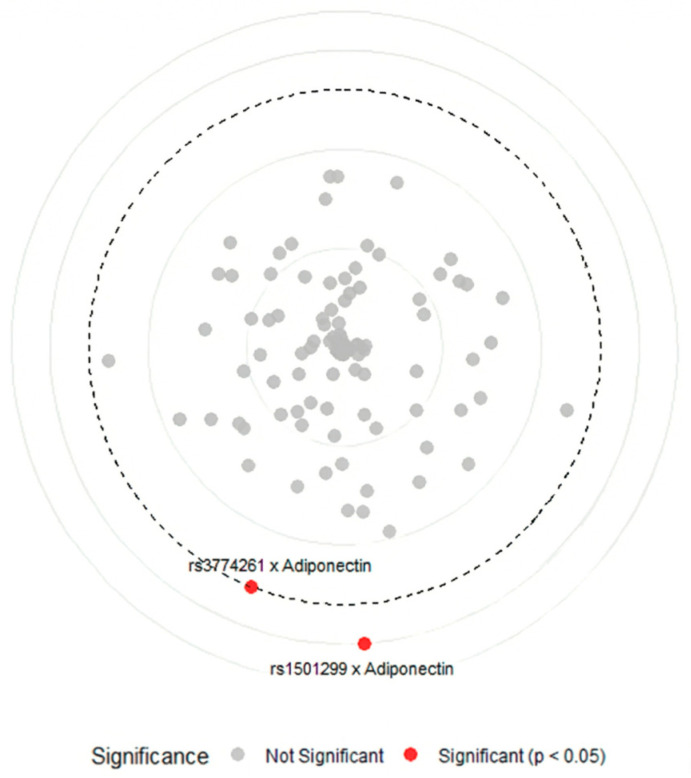
Circular Manhattan plot of gene–phenotype interactions between *ADIPOQ* variants and metabolic traits in obese women with PCOS. Points represent gene–environment interactions between *ADIPOQ* variants and clinical, biochemical, and hormonal variables, red points indicate significant interactions (−log_10_ Bonferroni *p*-value > 2.48) between rs3774261 and rs1501299 with adiponectin serum level, grey points indicate non-significant interactions (−log_10_ Bonferroni *p*-value < 2.48) between nine *ADIPOQ* variants with age, fasting glucose, HbA1c, free insulin, HOMA-IR, cholesterol, triglycerides, HDL, LDL, FSH, LH, progesterone, estradiol, and testosterone, and the bold dashed circular line marks the significance threshold (−log_10_ Bonferroni *p*-value = 2.48). HbA1C: glycated hemoglobin, HOMA-IR: homeostatic model assessment of insulin resistance, HDL: high-density lipoprotein, LDL: low-density lipoprotein, FSH: follicle-stimulating hormone, LH: luteinizing hormone.

To explore potential interactions between *ADIPOQ* variants and clinical or biochemical parameters, we conducted gene–environment interaction model using a Circular Manhattan plot approach (Figure 4). The plot illustrates the significance of interaction tests, with each point representing a tested variant–trait combination and the distance from the center indicating interaction strength. Points that cross the dashed significance threshold (−log_10_ Bonferroni *p* = 2.48) represent statistically significant interactions after correction for multiple testing.

The analysis revealed a notable specificity: among all tested clinical parameters, including markers of glucose metabolism (fasting glucose, HbA1c, insulin, and HOMA-IR), lipid profiles (cholesterol, triglycerides, HDL, and LDL), and reproductive hormones (FSH, LH, progesterone, estradiol, and testosterone), only serum adiponectin showed significant interactions with *ADIPOQ* genetics. Specifically, variants rs1501299 and rs3774261 showed significant gene–adiponectin interactions (red points) above the Bonferroni threshold (−log_10_ Bonferroni *p*-value > 2.48), while all other variant–trait combinations remained non-significant (grey points) (−log_10_ Bonferroni *p*-value < 2.48). These findings suggest that the genetic effects of *ADIPOQ* variants on obesity are not uniformly influenced by the metabolic environment but are specifically associated with circulating adiponectin levels.

### 3.7. Genotypes–Adiponectin Level Relationships

**Figure 5:**
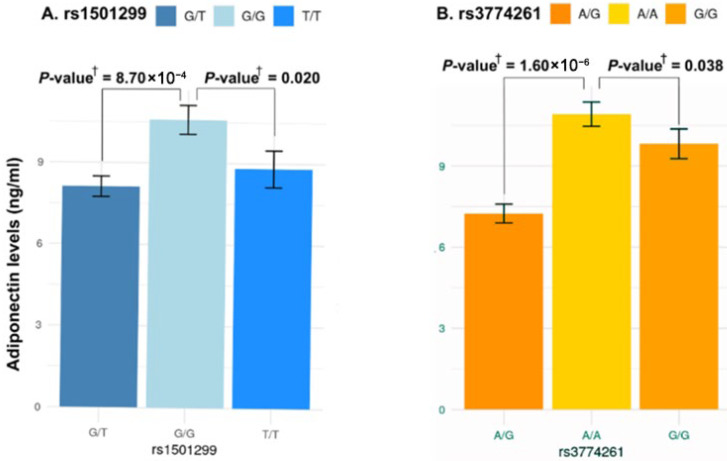
Association between the three genotypes of *ADIPOQ* rs1501299 and rs3774261 variants and serum adiponectin levels in obese women with PCOS. Each bar represents the mean serum adiponectin concentration (ng/mL) for each genotype, and error bars indicate the standard error of the mean. (**A**) Comparison of adiponectin levels across the three genotypes of rs1501299. (**B**) Comparison of adiponectin levels across the three genotypes of rs3774261. **^†^** *p*-values calculated using the Mann–Whitney U test.

To examine whether the identified obesity-risk variants rs1501299 and rs3774261 influence adiponectin concentration in obese women with PCOS, we compared adiponectin levels across genotypes (Figure 5). For rs1501299, homozygous (T/T) carriers of the risk allele (T) and heterozygous (G/T) had the lowest adiponectin levels compared to homozygous wild-type allele (G/G) carriers (*p* = 8.70 × 10^−4^ and *p* = 0.020, respectively) which indicates that the presence of the risk allele T is associated with a significant decrease in adiponectin levels (Figure 5A). Similarly, for rs3774261, the risk allele G showed a clear phenotypic effect. Individuals carrying the G allele (both homozygous (G/G) and heterozygous (A/G)) displayed a significant reduction in adiponectin levels compared to homozygous A/A wild-type individuals (*p* = 0.038 and *p* = 1.60 × 10^−6^, respectively) (Figure 5B).

### 3.8. Allele and Haplotype Effects

**Figure 6:**
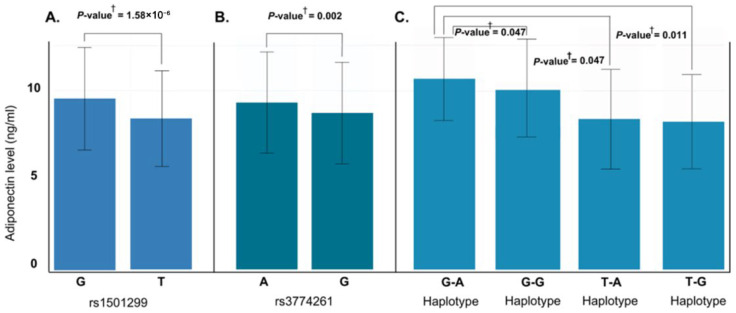
Effect of *ADIPOQ* rs1501299 and rs3774261 risk alleles and haplotypes on serum adiponectin levels in obese women with PCOS. (**A**) Allele-specific analysis for rs1501299: the risk allele T is associated with significantly lower adiponectin compared to the major allele G (*p* = 1.58 × 10^−6^). (**B**) Allele-specific analysis for rs3774261: the risk allele G is associated with significantly reduced adiponectin compared to the major allele A (*p* = 0.002). (**C**) Comparison of adiponectin levels across the four haplotypes of rs1501299 and rs3774261: The TG haplotype is associated with the lowest mean adiponectin levels compared to the GA haplotype. ^†^ *p*-values calculated using the Mann–Whitney U test.

**Figure 7:**
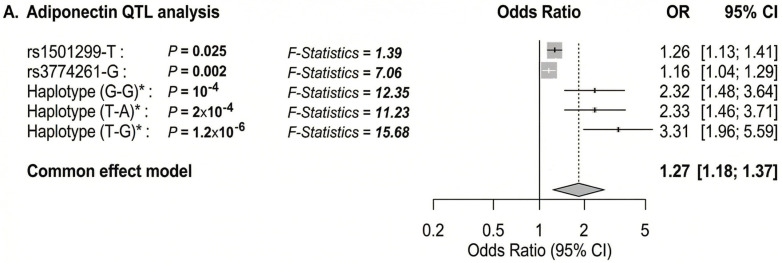

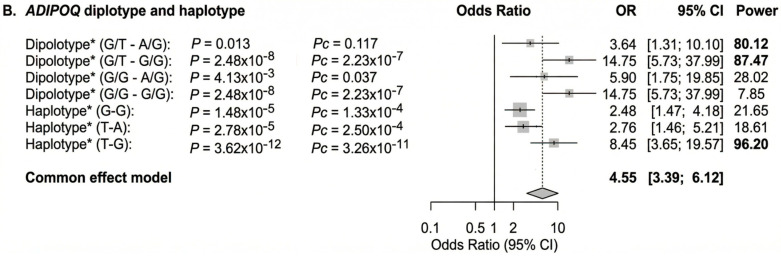

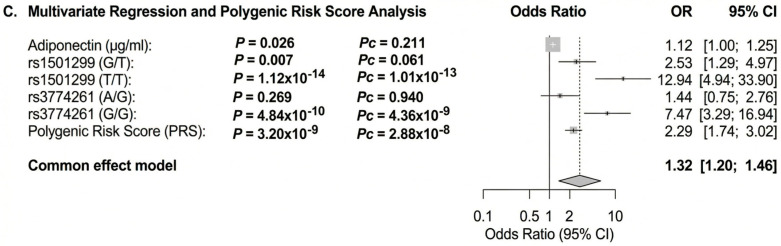
Forest plot showing the OR for the association of adiponectin and *ADIPOQ* rs1501299 and rs3774261 variants with obesity risk in women with PCOS. The forest plots were generated using “meta-R package (version 8.2-1)”. Squares and horizontal lines correspond to OR and 95% CI, respectively. The grey diamond represents the pooled (common-effect) odds ratio, and its width indicates the 95% confidence interval for the pooled estimate.. The unbroken vertical line is at the null value (OR = 1.0). The broken vertical line is at the average OR value in the common effect model. (**A**) Binary logistic regression association of diplotype and haplotype of *ADIPOQ* rs1501299 and rs3774261 variants with PCOS obesity. (**B**) Adiponectin QTL association with PCOS obesity. (**C**) Multivariate logistic regression showing independent associations of adiponectin levels, *ADIPOQ* variants rs1501299and rs3774261 (heterozygous and homozygous mutant genotypes) and a polygenic risk score (PRS) with PCOS obesity. * Diplotype and haplotype order: rs1501299 (G > T) and rs3774261 (G > A); OR: odds ratio; Pc: Bonferroni-corrected p values are generated by logistic regression carried out using SHEsisPlus software (http://shesisplus.bio-x.cn/SHEsis.html, accessed on 26 July 2025); after comparison with common diplotype G/G-A/A and common haplotype G-A.

To further characterize genetic associations, we performed allele-wise and haplotype-based analysis of rs1501299 and rs3774261 (Figure 6). Consistent with the genotype models, the specific risk alleles, (T) for rs1501299 and (G) for rs3774261, were significantly associated with reduced adiponectin levels compared to their respective wild-type alleles (Figure 6A,B). Haplotype analysis revealed an additive effect with individuals carrying the haplotypes combining both risk alleles exhibiting the lowest adiponectin levels, significantly lower than those with the wild-type G-A haplotype (*p* = 0.011) (Figure 6C). These findings suggest additive contributions of the two variants to the reduction in adiponectin levels. Given this significant impact on adiponectin, we proceeded with a quantitative trait locus (QTL) analysis to determine whether these specific alleles and haplotypes directly translate into increased obesity risk (Figure 7A). The results showed that both individual risk alleles (rs1501299-T and rs3774261-G) and their combined haplotypes were significantly associated with increased obesity risk. Notably, haplotypes carrying both risk alleles demonstrated the strongest associations, with the TG haplotype showing an odds ratio of 3.31 (95% CI: 1.96–5.59, *p* = 1.2 × 10^−6^), substantially exceeding the effects of single alleles. The overall common effect estimate (OR = 1.27, 95% CI: 1.18–1.37) confirms that these adiponectin-related genetic variants function as obesity susceptibility loci in PCOS. These results establish a clear link between the genetically determined reduction in adiponectin levels and clinical obesity outcomes.

### 3.9. Diplotype and Multivariable Modeling

To integrate genetic and biochemical determinants into a comprehensive model, we conducted diplotype analysis and multivariable logistic regression. In the diplotype analysis, individuals with combined risk diplotypes, specifically the double heterozygous (G/T-A/G) and with heterozygous/homozygous risk diplotype (G/T-G/G), exhibited significantly increased odds ratios for obesity compared to the reference wild-type diplotypes (G/G-A/A). The associations were significant (power > 0.8) with OR of 3.64 and 14.75, respectively (Figure 7B).

In multivariable regression, adiponectin levels, rs1501299, rs3774261, and the polygenic risk factor (PRS) remained significant independent predictors of obesity status. Notably, the combined model demonstrated superior predictive value compared to any single factor alone, emphasizing the complementary roles of biochemical and genetic determinants in the pathophysiology of obesity in PCOS (Figure 7C).

## 4. Discussion

Adiponectin plays an important role in maintaining metabolic homeostasis [17]. Unlike most factors derived from adipocytes, its circulating levels are inversely correlated with obesity with lower levels (hypoadiponectinemia) observed in obese individuals [13]. Clinically, low adiponectin is strongly associated with metabolic syndrome, IR, T2D, and cardiovascular disease [13]. In PCOS, a condition where IR and chronic inflammation frequently occur, reduced adiponectin levels are associated with metabolic disturbances, and contribute to both metabolic and reproductive abnormalities [26,34].

Given adiponectin’s central role in the metabolic process, variants of the *ADIPOQ* gene were investigated for their potential contribution to PCOS. However, findings have been inconsistent, likely due to differences in study design, population genetics, and phenotype definitions [2,35]. This study aimed to explore the role of nine variants in the *ADIPOQ* gene in relation to PCOS in a Tunisian cohort, with a specific interest in their association with hypoadiponectinemia and obesity.

Our results showed that, among women with PCOS, those classified as obese had significantly lower adiponectin levels compared to their normal-weight counterparts, which aligns with the established inverse relationship between adiposity and adiponectin [36]. It is noteworthy that the absolute adiponectin concentrations in our cohort are lower than those reported in some reference populations. Such discrepancies are common in adipokine research and likely attributable to methodological differences (e.g., specific ELISA kit sensitivity and calibration), population stratification (genetic background), and variations in BMI or metabolic severity across cohorts. Nonetheless, the relative difference, significantly lower levels in obese versus non-obese PCOS women, remains a significant finding consistent with the established pathophysiology of hypoadiponectinemia in obesity.

Genetically, we identified two variants, rs1501299 and rs3774261, as key *ADIPOQ* variants associated with obesity in patients with PCOS. Women carrying risk alleles at these loci showed substantially higher odds of obesity. For the intronic variant rs1501299 (also known as +276 G > T), the minor (T) allele demonstrated a dose-dependent effect: T/T homozygotes had over a five-fold increased risk of obesity compared to (G) allele carriers, while heterozygotes (G/T) had about a two-fold increased risk, indicating an additive effect of the (T) allele. Similarly, for rs3774261, located in the 3′ region of *ADIPOQ* gene, the G/G genotype (risk genotype) conferred approximately 3-fold higher odds of obesity compared to the (A/G) genotype. These associations remained significant after Bonferroni correction and multivariate adjustment, and were associated specifically with adiponectin concentrations, suggesting a relationship between genotype, adiponectin levels, and clinical phenotypes. No other *ADIPOQ* variants showed significant association with obesity in our population, underscoring rs1501299 and rs3774261 as key genetic determinants of obesity in the Tunisian population.

The identification of rs1501299 as a significant variant aligns with findings from previous studies. Li et al. (2011) [25] and Alfaqih et al. (2018) [24] suggested a protective role for the rs1501299 T allele in Asian and Jordanian populations, respectively. In contrast, our data indicate that the T allele of rs1501299 is significantly associated with an increased risk of obesity among Tunisian women with PCOS, suggesting that its effect may be specific to this phenotype and population. Such discrepancies may reflect differences in phenotype definition (PCOS susceptibility versus obesity severity in PCOS) as well as population-specific genetic architectures and environmental factors. This context-dependent effect highlights the complexity of PCOS genetics, where a variant can influence both syndrome incidence and specific sub-phenotypes in opposing ways. Our gene–phenotype interaction analysis supports this interpretation, indicating that the association of the rs1501299 (T) allele is largely related to adiponectin levels, which are, in turn, associated with obesity risk in PCOS patients. The interplay between genetic factors and environmental conditions or genetic epistasis may further modify the overall association of this variant with PCOS risk versus obesity severity across different populations.

As for rs3774261, fewer PCOS-specific data exist; however, our findings are indirectly supported by the study conducted by Ezzidi et al., 2020 [37], who examined multiple *ADIPOQ* variants in a Saudi population, and identified haplotypes containing rs3774261 that are associated with increased PCOS risk and contribute independently to obesity. This suggests that the rs3774261 (G) allele might not exert a significant effect alone, but when combined with other alleles, like rs1501299 (G) in the Saudi population or rs1501299 (T) in our study, it contributes to the overall risk. These findings align with earlier studies investigating *ADIPOQ* haplotypes in PCOS. Radavelli-Bagatini et al. (2013) [38] reported in a South Brazilian cohort that a specific haplotype, combining variants in exon 2 (45T > G) and intron 2 (276G > T) of the adiponectin gene, was associated with increased PCOS susceptibility. Notably, the haplotype carrying the 45T and 276G alleles was linked to higher PCOS risk. This observation aligns with our results, where individuals carrying both risk alleles exhibited the most severe metabolic outcomes. While Radavelli-Bagatini’s study focused primarily on PCOS susceptibility, our analysis extended this by examining the impact of haplotypes on obesity within PCOS patients. In our study, the combination of risk alleles at rs1501299 and rs3774261 was associated with the lowest adiponectin levels and the highest odds of obesity, with diplotype odds ratio approaching 15, though with wide confidence intervals due to small subgroup sizes.

This consistency across studies underscores the role of *ADIPOQ* genetic variation in PCOS, although its effects may differ depending on whether the focus is on disease susceptibility or metabolic sub-phenotypes (e.g., obese vs. non-obese PCOS patients).

These findings underscore the importance of analyzing haplotypes and gene–gene interactions, as such approach can reveal associations that single-variant analyses might overlook due to locus interdependence [27]. Conversely, some studies have reported no significant associations between common *ADIPOQ* variants and PCOS, emphasizing the heterogeneity of the condition. For instance, Escobar-Morreale et al. (2006) [26] found no significant differences in *ADIPOQ* variants (including 45T > G) between PCOS patients and controls in a Spanish cohort. Similarly, Xita et al. (2005) [27] observed that while the 45T > G and 276G > T variants were not associated with PCOS diagnosis in Greek women, they did influence circulating adiponectin levels and indices of IR.

Discrepancies among studies likely result from a combination of factors, including ethnic variations in allele frequencies, small sample sizes that lead to an increase in the risk of false negatives or inconsistent findings, differing diagnostic criteria for PCOS, and variable linkage disequilibrium patterns with potential causal variants. Our study contributes to this growing body of evidence by offering a comprehensive analysis in an understudied population (North African Tunisians), using an adequate overall sample size for detecting significant genetic findings and employing rigorous statistical corrections.

The persistent significance of rs1501299 across multiple studies, despite variations in its association with PCOS risk, reinforces its status as a real locus of interest. Simultaneously, the identification of rs3774261 as a significant factor in our analysis highlights a novel avenue for future research, as this variant has been largely unexplored in the context of PCOS.

Beyond genetic association, these variants may also have clinical implications for treatment response. For example, response to dietary restrictions/changes is associated with *ADIPOQ* genotypes; for example, subjects with the GG genotype of an *ADIPOQ* rs1501299 gene variant showed better improvement in adiponectin levels and metabolic profile compared to T allele carriers [39] in obese subjects who also showed lack of response to hypocaloric diet if they carried the T allele of *ADIPOQ* gene variant (rs1501299) [40] while dietary approaches to stop hypertension (DASH) only lowered hypertension in patients with TT genotype [41]. This is particularly interesting in the context of PCOS, where patients respond differently to the first-line treatment of weight management. It would thus be interesting to investigate whether *ADIPOQ* genotype modulates the efficacy of dietary interventions in women with PCOS.

Our study has several strengths. We used comprehensive analysis including multiple methods like binary, haplotype, diplotype, epistasis, and multivariable modelling. This methodology provided strong evidence for the roles of rs1501299 and rs3774261 variants in PCOS. By targeting the Tunisian population (a North African group), we added a novel insight from a less-studied population, helping expand the global understanding of PCOS genetics.

From a clinical perspective, these findings underscore the potential utility of *ADIPOQ* genotyping as part of a precision medicine approach in PCOS management. The identification of high-risk carriers, particularly those with recessive genotypes of rs1501299 and rs3774261, may help clinicians identify a subgroup of women with PCOS who are genetically predisposed to severe hypoadiponectinemia and obesity. For these patients, early and aggressive lifestyle interventions or pharmacological strategies aimed at enhancing insulin sensitivity and adiponectin levels (e.g., PPAR gamma agonists) could be particularly beneficial. Furthermore, integrating these genetic markers with traditional biochemical profiles could refine risk stratification algorithms, allowing for more personalized surveillance of metabolic complications in this high-risk population.

We also recognize some limitations. First, cross-sectional study design (did not include a non PCOS-group) precludes establishing temporal or causal relationships between *ADIPOQ* variants, adiponectin levels, and obesity. Second, the lack of a non-PCOS control group limits our ability to determine if observed associations are specific to PCOS or reflect general BMI-related metabolic effects. Third, while our overall sample size is adequate for detecting the main effects of common genetic variants and represents one of the larger studies in North African populations, certain rare genotype combinations and specific interaction analyses were limited by small subgroup sizes. Additionally, lifestyle factors such as diet and physical activity, which are known to influence adiponectin levels and obesity risk, were not collected in this cohort and thus could not be adjusted for in the multivariate analysis. We also acknowledge potential population stratification, as our study was restricted to Tunisian women of North African ancestry; replication in diverse ethnic populations is essential. Furthermore, our polygenic risk score (PRS) requires external validation in independent cohorts to confirm its predictive utility. Additionally, we excluded women with extreme BMI values (<20 or >40 kg/m^2^), limiting generalizability to these groups. Finally, we measured total rather than high molecular-weight adiponectin, which may have distinct metabolic relevance. Future longitudinal studies with multi-ethnic cohorts are needed to confirm these associations and elucidate the functional impact of three variants.

## 5. Conclusions

Our study provides evidence that the *ADIPOQ* variants rs1501299 and rs3774261 are significantly associated with obesity in women with PCOS. Importantly, we demonstrate that this association is substantially related to hypoadiponectinemia, suggesting a biological pathway linking genetic variants to obesity in PCOS. These findings not only bridge a critical gap in understanding the genetic associations with metabolic dysfunction in PCOS but also underscore the potential of *ADIPOQ* genotyping for personalized risk stratification, paving the way for novel therapeutic strategies targeting the adiponectin pathway in this prevalent endocrine disorder.

## Figures and Tables

**Table 1:** Clinical characteristics of women study subjects with PCOS.

	Non-Obese Women with PCOS (n = 238)	Obese Women with PCOS (n = 86)		
	Median (25th–75th)	Median (25th–75th)	*p* ^a^	*p* ^b^
Age (year)	28 (24–32)	28 (24.25–30)	0.508	0.782
BMI (kg/m^2^)	25.91 (23.65–27.52)	32.47 (30.83–35.97)	<0.001	<0.001
FPG (mmol/L)	8 (5.9–9.47)	7.50 (5.35–9.62)	0.662	0.827
HbA1c (%)	5.30 (4.92–5.60)	5.40 (5.10–5.70)	0.066	0.332
FINS (µU/mL)	14.10 (9.32–18.25)	12.44 (9.03–18.20)	0.499	0.782
HOMA-IR	4.90 (2.47–7.35)	4.40 (2.36–6.51)	0.227	0.543
Cholesterol (mmol/L)	5.20 (4.5–6.2)	5.20 (4.2–6.45)	0.521	0.782
Triglycerides (mmol/L)	1.47 (1.20–2.06)	1.38 (1.01–2.02)	0.157	0.543
HDL (mmol/L)	1.32 (1.12–1.60)	1.20 (0.96–1.42)	0.040	0.310
LDL (mmol/L)	3.32 (2.57–4.56)	3.24 (2.59–4.46)	0.742	0.856
FSH (IU/L)	5.50 (2.76–6.77)	5.40 (1.29–6.98)	0.656	0.827
LH (IU/L)	4.11 (0.92–9.29)	2.11 (0.25–7.74)	0.213	0.543
Progesterone (ng/mL)	19.80 (1.67–20.19)	19.80 (1.89–79.17)	0.253	0.543
Estradiol (pg/mL)	66.87 (46.05–136.41)	66.74 (47.25–111.85)	0.970	0.970
Testosterone (ng/dL)	62 (45.25–91.75)	62 (43–90)	0.955	0.970
Adiponectin (μg/mL)	9.81 (7.42–11.81)	8.85 (6.71–11.5)	0.004	0.030

Abbreviations: BMI: body mass index; PCOS: Polycystic Ovary Syndrome; non-obese women with PCOS (BMI: 20–29.99 kg/m^2^); obese women with PCOS (BMI: 30–39.99 kg/m^2^); FPG: fasting plasma glucose; FINS: fasting plasma insulin, HbA1C: glycated hemoglobin; HOMA-IR: homeostatic model assessment of insulin resistance;, HDL: high-density lipoprotein; LDL: low-density lipoprotein; FSH: follicle-stimulating hormone; LH: luteinizing hormone; ^a^ Wilcoxon *p*-value; ^b^ FDR-adjusted *p*-value.

**Table 2:** Binary logistic regression of clinical variables with PCOS obesity.

	AUC	*p*-Value ^a^	*p*-Value ^b^	OR (95% CI)	AIC	Cohen’s d	Power
Age (year)	0.476	0.335	0.998	0.97 (0.93–1.03)	372.44	0.121	0.174
FPG (mmol/L)	0.516	0.655	1.000	0.96 (0.88–1.09)	382.99	0.056	0.723
HbA1c (%)	0.567	0.194	0.968	1.30 (0.87–1.93)	377.88	0.164	0.271
FINS (µU/mL)	0.525	0.492	1.000	0.99 (0.95–1.03)	382.27	0.086	0.105
HOMA-IR	0.544	0.387	1.000	0.96 (0.89–1.04)	377.43	0.109	0.138
Cholesterol (mmol/L)	0.477	0.956	1.000	0.99 (0.83–1.19)	375.32	0.007	0.503
Triglycerides (mmol/L)	0.551	0.394	1.000	0.87 (0.64–1.19)	381.43	0.107	0.136
HDL (mmol/L)	0.601	0.005	0.075	2.84 (1.38–5.86)	373.74	0.364	0.815
LDL (mmol/L)	0.488	0.375	1.000	1.08 (0.90–1.30)	370.60	0.111	0.143
FSH (IU/L)	0.516	0.814	1.000	0.99 (0.95–1.04)	374.88	0.030	0.563
LH (IU/L)	0.545	0.769	1.000	0.99 (0.96–1.03)	378.61	0.037	0.598
Progesterone (ng/mL)	0.541	0.222	0.982	1.00 (0.99–1.01)	380.76	0.154	0.231
Estradiol (pg/mL)	0.520	0.678	1.000	1.00 (0.99–1.01)	381.29	0.057	0.739
Testosterone (ng/dL)	0.502	0.822	1.000	1.00 (0.99–1.01)	383.36	0.028	0.557
Adiponectin (μg/mL)	0.605	3.75 × 10^−4^	0.006	1.15 (1.05–1.27) ^c^	364.28	0.366	0.823

Abbreviations: FPG: fasting plasma glucose; FINS: fasting insulin; HbA1C: glycated hemoglobin; HOMA-IR: homeostatic model assessment of insulin resistance; HDL: high-density lipoprotein; LDL: low-density lipoprotein; FSH: follicle-stimulating hormone; LH: luteinizing hormone; AUC: Area under the receiver operating characteristic (ROC) curve; OR (95% CI): odds ratio (95% confidence interval); AIC: akaike information criterion was used to determine the best model of association for each clinical, biochemical and hormonal variable (the lower the better the model is); Cohen’d: minimum effect size d coefficient (<0.2: small effect, 0.2–0.8: moderate effect, >0.8: large effect); ^a^ Pearson Chi-square nominal *p*-value; ^b^ FDR-adjusted *p*-value; ^c^: OR > 1 for adiponectin reflect the inverse relationship between adiponectin levels and obesity risk. Hypoadiponectinemia is associated with a higher odds ratio for obesity.

**Table 3:** *ADIPOQ* alternate allele and genotype frequencies analysis.

	Genotype and Allele Distributions: n (%)		
Variant	All Women with PCOS (n = 324)	Non-Obese Women with PCOS (n = 238)	Obese Women with PCOS (n = 86)
rs16861194 (A < G)			
A/A	280 (86.42)	210 (88.23)	69 (80.23)
A/G	40 (12.34)	25 (10.50)	15 (17.44)
G/G	4 (1.23)	3 (1.26)	2 (2.32)
Alt. Allele (G)	48 (7.41)	31 (6.51)	19 (11.05)
HWE-*p*-value ^a^	0.072		
rs17300539 (G < A)			
G/G	295 (91.05)	213 (89.50)	82 (95.35)
G/A	27 (8.33)	23 (9.66)	4 (4.65)
A/A	2 (0.62)	2 (0.84)	0
Alt. Allele (A)	31 (4.78)	27 (5.67)	4 (2.32)
HWE-*p*-value ^a^	0.125		
rs266729 (C < G)			
C/C	186 (57.41)	143 (60.08)	43 (50.00)
C/G	120 (37.04)	82 (34.45)	38 (44.19)
G/G	18 (5.55)	13 (5.46)	5 (5.81)
Alt. Allele (G)	156 (24.07)	108 (22.69)	48 (27.91)
HWE-*p*-value ^a^	0.813		
rs822395 (A < C)			
A/A	212 (65.43)	156 (65.55)	56 (65.12)
A/C	102 (31.48)	76 (31.93)	26 (30.23)
C/C	10 (3.08)	6 (2.52)	4 (4.65)
Alt. Allele (C)	122 (18.83)	88 (18.49)	34 (19.77)
HWE-*p*-value ^a^	0.589		
rs822396 (A < G)			
A/A	286 (88.27)	210 (88.23)	76 (88.37)
A/G	36 (11.11)	26 (10.92)	10 (11.63)
G/G	2 (0.62)	2 (0.84)	0
Alt. Allele (G)	40 (6.17)	30 (6.30)	10 (5.81)
HWE-*p*-value ^a^	0.463		
rs2241766 (T < G)			
T/T	206 (63.58)	150 (63.02)	56 (65.12)
T/G	106 (32.72)	78 (32.77)	28 (32.56)
G/G	12 (3.70)	10 (4.20)	2 (2.32)
Alt. Allele (G)	130 (20.06)	98 (20.59)	32 (18.60)
HWE-*p*-value ^a^	0.719		
rs2241767 (A < G)			
A/A	204 (62.96)	146 (61.34)	58 (67.44)
A/G	106 (32.72)	82 (34.45)	24 (27.91)
G/G	14 (4.32)	10 (4.20)	4 (4.65)
Alt. Allele (G)	134 (20.68)	102 (21.43)	32 (18.60)
HWE-*p*-value ^a^	0.961		
rs1501299 (G < T)			
G/G	143 (44.13)	116 (48.74)	27 (31.39)
G/T	151 (46.60)	111 (46.64)	40 (46.51)
T/T	30 (9.26)	11 (4.62)	19 (22.09)
Alt. Allele (T)	211 (32.56)	133 (27.94)	78 (45.35)
HWE-*p*-value ^a^	0.271		
rs3774261 (A < G)			
A/A	134 (41.36)	110 (46.21)	24 (27.90)
A/G	136 (41.97)	100 (42.01)	36 (41.86)
G/G	54 (16.67)	28 (11.76)	26 (30.23)
Alt. Allele (G)	244 (37.65)	156 (32.77)	88 (51.16)
HWE-*p*-value ^a^	0.056		

Abbreviation: All women with PCOS (BMI: 20–39.99 kg/m^2^); non-obese women with PCOS (BMI: 20–29.99 kg/m^2^); obese women with PCOS (BMI: 30–39.99 kg/m^2^); Alt. Allele: alternate allele (data available in https://www.ncbi.nlm.nih.gov/snp/, accessed on 25 July 2025); HWE-*p*-value ^a^: Hardy–Weinberg equilibrium *p*-value (Pearson Chi-square test).

**Table 4:** Binary logistic regression association of *ADIPOQ* variants with PCOS obesity.

Obese Women with PCOS (n = 86) vs. Non-Obese Women with PCOS (n = 238)						
**Variant**	Inheritance Model	*p*-Value ^a^	*p*-Value ^b^	OR (95% CI) ^c^	AIC	Power
rs16861194	Additive	0.078	0.698	1.67 (0.93–2.93)	376	0.294
	Codominant	0.086	0.545	1.83 (0.91–3.66)	370.6	
	Dominant	0.074	0.499	1.85 (0.95–3.58)	368.6	
	Recessive	0.510	0.998	1.87 (0.31–11.36)	372.1	
rs17300539	Additive	0.102	0.918	0.42 (0.12–1.06)	375.6	0.194
	Codominant	0.144	0.753	0.45 (0.15–1.35)	371	
	Dominant	0.083	0.541	0.42 (0.14–1.23)	369.6	
	Recessive	0.994	1.00	NA	371.5	
rs266729	Additive	0.169	1.00	1.33 (0.88–1.98)	377.1	0.171
	Codominant	0.098	0.605	1.54 (0.92–2.58)	371.8	
	Dominant	0.106	0.653	1.50 (0.92–2.47)	370.1	
	Recessive	0.903	1.00	1.07 (0.37–3.09)	372.7	
rs822395	Additive	0.709	1.00	1.09 (0.69–1.69)	378.8	0.061
	Codominant	0.861	1.00	0.95 (0.55–1.63)	373.6	
	Dominant	0.943	1.00	1.02 (0.61–1.71)	372.7	
	Recessive	0.348	0.977	1.89 (0.52–6.85)	371.6	
rs822396	Additive	0.823	1.00	0.92 (0.42–1.84)	378.9	0.049
	Codominant	0.878	1.00	1.06 (0.49–2.31)	373.7	
	Dominant	0.973	1.00	0.99 (0.46–2.13)	372.7	
	Recessive	0.999	1.00	NA	371.7	
rs2241766	Additive	0.574	1.00	0.88 (0.55–1.36)	378.6	0.064
	Codominant	0.885	1.00	0.96 (0.57–1.63)	374	
	Dominant	0.730	0.999	0.91 (0.54–1.53)	372.4	
	Recessive	0.408	0.991	0.54 (0.12–2.53)	372.2	
rs2241767	Additive	0.434	1.00	0.84 (0.53–1.29)	378.3	0.080
	Codominant	0.273	0.943	0.74 (0.43–1.27)	372.5	
	Dominant	0.313	0.966	0.77 (0.45–1.29)	371.1	
	Recessive	0.861	1.00	1.11 (0.34–3.64)	372.6	
rs1501299 ^d^	Additive	2.90 × 10^−5^	2.61 × 10^−4^	2.32 (1.57–3.48)	360.6	0.831
	Codominant	0.120	0.683	1.57 (0.90–2.75)	355.4	
	Dominant	0.005	0.044	2.07 (1.22–3.50)	365.1	
	Recessive	7.93 × 10^−6^	7.14 × 10^−5^	5.18 (2.32–11.56)	356	
rs3774261 ^d^	Additive	7.34 × 10^−5^	6.60 × 10^−4^	2.03 (1.43–2.89)	362.7	0.851
	Codominant	0.091	0.576	1.59 (0.88–2.87)	359.3	
	Dominant	0.003	0.027	2.13 (1.24–3.67)	364.8	
	Recessive	1.69 × 10^−4^	1.52 × 10^−3^	3.13 (1.69–5.78)	359.8	

Abbreviation: Non-obese women with PCOS (BMI: 20–29.99 kg/m^2^); obese women with PCOS (BMI: 30–39.99 kg/m^2^); NA: not applicable; AIC: akaike information criterion was used to determine the best model of inheritance for each variant (the lower the better the model is); ^a^ Pearson Chi-square nominal *p*-value; ^b^ Bonferroni-corrected *p*-value (adjusted for the total number of variants under each genetic model); ^c^ OR (95% CI): adjusted odds ratio (95% confidence interval) for age, LH, FSH, progesterone, estradiol, and testosterone; ^d^ Cramer’s V: rs1501299 = 0.278 (medium) and rs3774261 = 0.236 (medium).

## Data Availability

The original contributions presented in this study are included in the article/Supplementary Materials. Further inquiries can be directed to the corresponding authors.

## Supplementary Materials

The following supporting information can be downloaded at https://www.mdpi.com/article/10.3390/life16010024/s1, Figure S1: Manhattan plot for Shapiro-Wilk normality test of clinical, biochemical and hormonal variables of PCOS women; Table S1: Multivariate Linear Regression Analysis Examining the Interaction of Clinical Variables with BMI Range (20.00–29.99 kg.m^−2^) among PCOS Cases; Table S2: Multivariate Linear Regression Analysis Examining the Interaction of ADIPOQ Variants, under Additive Model, with BMI Range (20.00–29.99 kg.m^−2^) among PCOS cases.
