# Supplementary material for: ADIPOQ Variants rs1501299 and rs3774261 and Hypoadiponectinemia in Obese Women with PCOS: Genetic and Metabolic Interactions
Source: Life (Basel). 2025 Dec 23;16(1):24. doi: 10.3390/life16010024 (PMC12843212; doi:10.3390/life16010024)
Supplement: Supplementary file 1 [file life-16-00024-s001.zip › life-4014590-supplementary.pdf]

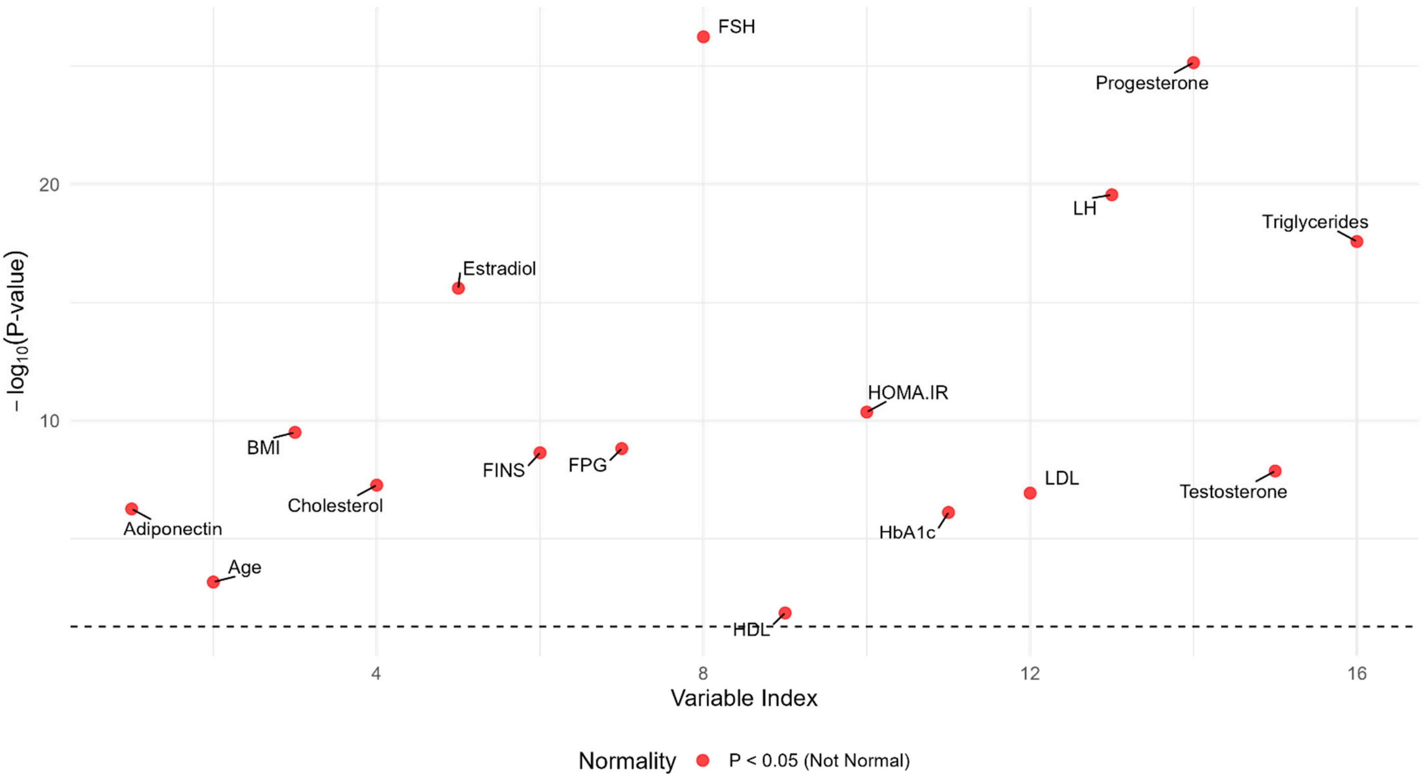

Supplementary Figure S1. Manhattan plot for Shapiro-Wilk normality test of clinical, biochemical and hormonal variables of women study subjects with PCOS

BMI: body mass index, FPG: fasting plasma glucose, FINS: fasting plasma insulin, HbA1C: glycated hemoglobin, HOMA-IR: homeostatic model assessment of insulin resistance, HDL: high-density lipoprotein, LDL: low-density lipoprotein, FSH: follicle-stimulating hormone, LH: luteinizing hormone

Supplementary Table S1. Clinical characteristics of women study subjects with PCOS

	**non-obese PCOS (n = 238)**	**Obese PCOS (n = 86)**		
	Median (25th-75th)	Median (25th-75th)	*P* †	*P* ⸸
Age (year)	28 (24-32)	28 (24.25-30)	0.508	0.782
BMI (Kg/m^2)	25.91 (23.65-27.52)	32.47 (30.83-35.97)	< 0.001	< 0.001
FPG (mmol/L)	8 (5.9-9.47)	7.50 (5.35-9.62)	0.662	0.827
HbA1c (%)	5.30 (4.92-5.60)	5.40 (5.10-5.70)	0.066	0.332
FINS (µU/mL)	14.10 (9.32-18.25)	12.44 (9.03-18.20)	0.499	0.782
HOMA-IR	4.90 (2.47-7.35)	4.40 (2.36-6.51)	0.227	0.543
Cholesterol (mmol/L)	5.20 (4.5-6.2)	5.20 (4.2-6.45)	0.521	0.782
Triglycerides (mmol/L)	1.47 (1.20-2.06)	1.38 (1.01-2.02)	0.157	0.543
HDL (mmol/L)	1.32 (1.12-1.60)	1.20 (0.96-1.42)	0.040	0.310
LDL (mmol/L)	3.32 (2.57-4.56)	3.24 (2.59-4.46)	0.742	0.856
FSH (IU/L)	5.50 (2.76-6.77)	5.40 (1.29-6.98)	0.656	0.827
LH (IU/L)	4.11 (0.92-9.29)	2.11 (0.25-7.74)	0.213	0.543
Progesterone (ng/mL)	19.80 (1.67-20.19)	19.80 (1.89-79.17)	0.253	0.543
Estradiol (pg/ml)	66.87 (46.05-136.41)	66.74 (47.25-111.85)	0.970	0.970
Testosterone (ng/dl)	62 (45.25-91.75)	62 (43-90)	0.955	0.970
Adiponectin (µg/ml)	9.81 (7.42-11.81)	8.85 (6.71-11.5)	0.004	0.030

BMI: body mass index, Obese PCOS (BMI: 30-39.99 Kg/m^2), non-obese PCOS (BMI: 20–29.99 Kg/m^2), FPG: fasting plasma glucose, FINS: fasting plasma insulin, HbA1C: glycated hemoglobin, HOMA-IR: homeostatic model assessment of insulin resistance, HDL: high-density lipoprotein, LDL: low-density lipoprotein, FSH: follicle-stimulating hormone, LH: luteinizing hormone

† Wilcoxon *P*-value,

⸸ FDR-adjusted *P*-value.

Supplementary Table S2. *ADIPOQ* alternate allele and genotype frequencies analysis

	Genotype and allele distributions: n (%)		
**Variant**	**All PCOS (n = 324)**	**non-obese PCOS (n = 238)**	**Obese PCOS (n = 86)**
**rs16861194 (A<G)**			
A/A	280 (86.42)	210 (88.23)	69 (80.23)
A/G	40 (12.34)	25 (10.50)	15 (17.44)
G/G	4 (1.23)	3 (1.26)	2 (2.32)
Alt. Allele (G)	48 (7.41)	31 (6.51)	19 (11.05)
HWE- *P*-value†	0.072		
**rs17300539 (G<A)**			
G/G	295 (91.05)	213 (89.50)	82 (95.35)
G/A	27 (8.33)	23 (9.66)	4 (4.65)
A/A	2 (0.62)	2 (0.84)	0
Alt. Allele (A)	31 (4.78)	27 (5.67)	4 (2.32)
HWE- *P*-value†	0.125		
**rs266729 (C<G)**			
C/C	186 (57.41)	143 (60.08)	43 (50.00)
C/G	120 (37.04)	82 (34.45)	38 (44.19)
G/G	18 (5.55)	13 (5.46)	5 (5.81)
Alt. Allele (G)	156 (24.07)	108 (22.69)	48 (27.91)
HWE- *P*-value†	0.813		
**rs822395 (A<C)**			
A/A	212 (65.43)	156 (65.55)	56 (65.12)
A/C	102 (31.48)	76 (31.93)	26 (30.23)
C/C	10 (3.08)	6 (2.52)	4 (4.65)
Alt. Allele (C)	122 (18.83)	88 (18.49)	34 (19.77)
HWE- *P*-value†	0.589		
**rs822396 (A<G)**			
A/A	286 (88.27)	210 (88.23)	76 (88.37)
A/G	36 (11.11)	26 (10.92)	10 (11.63)
G/G	2 (0.62)	2 (0.84)	0
Alt. Allele (G)	40 (6.17)	30 (6.30)	10 (5.81)
HWE- *P*-value†	0.463		
**rs2241766 (T<G)**			
T/T	206 (63.58)	150 (63.02)	56 (65.12)
T/G	106 (32.72)	78 (32.77)	28 (32.56)
G/G	12 (3.70)	10 (4.20)	2 (2.32)
Alt. Allele (G)	130 (20.06)	98 (20.59)	32 (18.60)
HWE- *P*-value†	0.719		
**rs2241767 (A<G)**			
A/A	204 (62.96)	146 (61.34)	58 (67.44)
A/G	106 (32.72)	82 (34.45)	24 (27.91)
G/G	14 (4.32)	10 (4.20)	4 (4.65)
Alt. Allele (G)	134 (20.68)	102 (21.43)	32 (18.60)
HWE- *P*-value†	0.961		
**rs1501299 (G<T)**			
G/G	143 (44.13)	116 (48.74)	27 (31.39)
G/T	151 (46.60)	111 (46.64)	40 (46.51)
T/T	30 (9.26)	11 (4.62)	19 (22.09)
Alt. Allele (T)	211 (32.56)	133 (27.94)	78 (45.35)
HWE- *P*-value†	0.271		
**rs3774261 (A<G)**			
A/A	134 (41.36)	110 (46.21)	24 (27.90)
A/G	136 (41.97)	100 (42.01)	36 (41.86)
G/G	54 (16.67)	28 (11.76)	26 (30.23)
Alt. Allele (G)	244 (37.65)	156 (32.77)	88 (51.16)
HWE- *P*-value†	0.056		

Obese (BMI: 30-39.99 Kg/m^2), non-obese PCOS (BMI: 20–29.99 Kg/m^2), Alt. Allele: Alternate allele (data available in https://www.ncbi.nlm.nih.gov/snp/),

HWE-*P*†: Hardy Weinberg equilibrium *P*-value (Pearson chi-square test).
